# EEG Suppression Associated with Apneic Episodes in a Neonate

**DOI:** 10.1155/2012/250801

**Published:** 2012-03-19

**Authors:** Evonne Low, Eugene M. Dempsey, C. Anthony Ryan, Janet M. Rennie, Geraldine B. Boylan

**Affiliations:** ^1^Neonatal Brain Research Group, Neonatal Intensive Care Unit, Cork University Maternity Hospital, Cork, Ireland; ^2^Department of Pediatrics and Child Health, University College Cork, Cork, Ireland; ^3^Elizabeth Garrett Anderson Wing, University College Hospital, London, UK

## Abstract

We describe the EEG findings from an ex-preterm neonate at term equivalent age who presented with intermittent but prolonged apneic episodes which were presumed to be seizures. A total of 8 apneic episodes were captured (duration 23–376 seconds) during EEG monitoring. The baseline EEG activity was appropriate for corrected gestational age and no electrographic seizure activity was recorded. The average baseline heart rate was 168 beats per minute (bpm) and the baseline oxygen saturation level was in the mid-nineties. Periods of complete EEG suppression lasting 68 and 179 seconds, respectively, were recorded during 2 of these 8 apneic episodes. Both episodes were accompanied by bradycardia less than 70 bpm and oxygen saturation levels of less than 20%. Short but severe episodes of apnea can cause complete EEG suppression in the neonate.

## 1. Introduction

Despite the frequency with which apnea occurs in the neonate and the concern about adverse long-term effects [1], few studies have examined the effects of apneic episodes simultaneously with recorded multichannel electroencephalography (EEG) [2]. Previous EEG studies in term neonates presenting with apnea but without an accompanying bradycardia have shown that seizures [3], particularly temporal lobe seizures [4], are a common etiology. The EEG changes associated with non-seizure apneic episodes in term neonates have not been described in detail. One study from 1969 has shown that apneic events during weaning from the ventilator in preterm neonates induced EEG suppression (<10 *μ*V) when oxygen partial pressures fell to approximately 20 mmHg [5]. Whether EEG suppression is a common occurrence during intermittent apneic episodes in neonates is not known and neither is the effect of the duration and severity of these events.

Sustained EEG suppression in the term neonate is a worrying sign and is often seen following the acute phase of moderate to severe hypoxic-ischemic encephalopathy [6]. EEG recovery can take hours or even days, depending on the severity of the primary injury and in very severe cases, the EEG may only recover with very low amplitude activity. In this case report, we were particularly interested in documenting the EEG changes which occur during intermittent episodes of hypoxia and bradycardia due to apnea in an ex-preterm neonate at term equivalent age.

## 2. Case Report

A female neonate was delivered by emergency Caesarean-section for maternal hypertension at 32 weeks (birthweight 1.9 kg (75th percentile)). At corrected gestational age of 38 weeks, she presented with apneic events associated with bradycardia and cyanosis. While being mechanically ventilated, she displayed some abnormal movements: hyperextension of the arms, jerking movements of all four limbs, thumb abduction, and hyperextension of the trunk during these apneic events. Prior to EEG monitoring, the neonate received intravenous phenobarbitone (10 mg/kg) and phenytoin (15 mg/kg) when clinical suspicion of seizures was raised. Cranial ultrasound imaging was normal. Chest radiograph showed right middle lobe consolidation secondary to viral bronchiolitis. The apneic events were attributed to intermittent mechanical obstruction of the endotracheal tube by copious secretions relating to bronchiolitis.

A NicOne digital video-EEG system (CareFusion NeuroCare, WI, USA) was used to record multichannel EEG in this neonate for a total of 22 hours, using scalp electrodes (F3, F4, C3, C4, T3, T4, O1, O2, and Cz). Continuous vital signs such as respiration, electrocardiogram (ECG), and oxygen saturations were monitored simultaneously using the IntelliVue MP70 Neonatal monitor (Philips, Boeblingen, Germany). The entire EEG recording was reviewed and annotated by an experienced neonatal neurophysiologist (GB). Apnea was defined as cessation of airflow for more than 20 seconds, or cessation of airflow for less than 20 seconds with bradycardia (20% below the baseline heart rate), or cessation of airflow for less than 20 seconds with oxygen desaturations below 80% [7]. Suppression of EEG activity to below 5 *μ*V in all EEG channels for at least 10 seconds was defined as complete EEG suppression.

Prior to gestational age of 38 weeks, the neonate did not have any apneic events. The background EEG activity prior to the apneic episodes showed continuous mixed frequency activity with the baseline EEG voltage ranging from 50 to 100 microvolts, which was appropriate for gestational age and electrographic seizure activity was not present before, during, or after the apneic events. The average baseline heart rate was 168 beats per minute (bpm), oxygenation saturations were in the mid-nineties and the neonate remained normotensive throughout monitoring.

**Figure 1:**
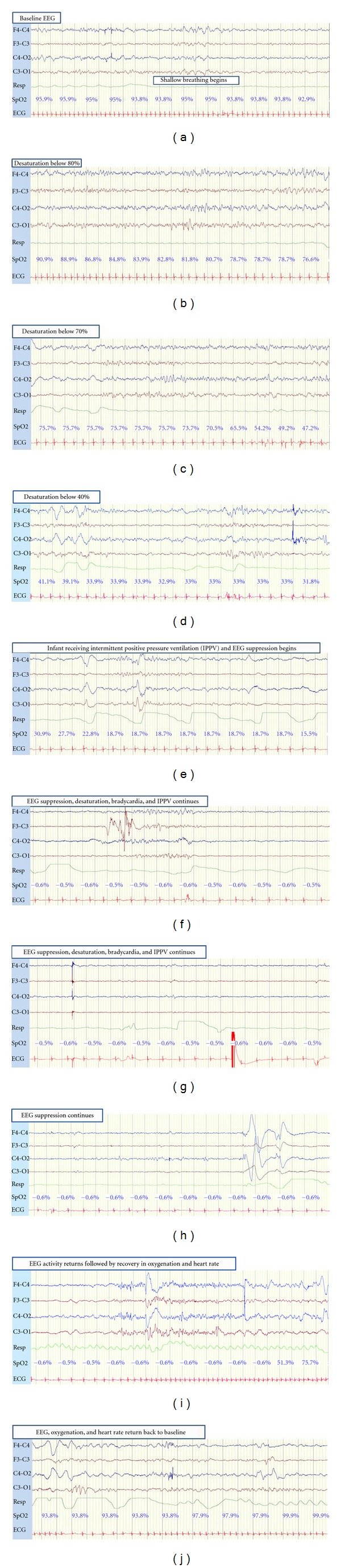
EEG recording showing the sequence of events evolving from baseline values associated with the first episode of complete EEG suppression. Calibration is 1 second and 50 microvolts.

The neonate had a total of eight apneic episodes during EEG monitoring, two of which required intermittent positive pressure ventilation, chest compressions, and adrenaline for recovery. Soon after the onset of both of these more prolonged apneic episodes (duration: 213 and 376 seconds resp.), there was a rapid decline in heart rate to 66 and 54 bpm, respectively, and oxygen saturation decreased to below 20% during both episodes which were accompanied with profound central cyanosis (Table 1). As heart rate and saturations declined, the EEG developed a burst suppression pattern. When the heart rate reached 66 and 54 bpm, respectively, and when oxygen saturations were below 20%, the EEG became completely suppressed. In both of these episodes, the EEG amplitude was completely suppressed for 68 and 179 seconds, respectively. During the recovery phase in both episodes, oxygen saturation improved to approximately 30 to 40% before EEG activity returned. Figures 1(a)–1(j) illustrate the sequence of events associated with the first episode of complete EEG suppression. The other six recorded apneic episodes in this neonate were less profound in duration (mean (range) = 79 (23–119) seconds) and were not accompanied by any EEG changes. The mean (range) of the lowest oxygen desaturation was 45 (24–69)% and the mean (range) of the lowest bradycardia was 99 (72–132) bpm.

## 3. Discussion

This case report has shown that episodes of transient but complete EEG suppression can occur during prolonged apneic episodes in the neonate particularly when they are accompanied by profound bradycardia and oxygen desaturation.

The apneic events in our case report were not associated with seizures on the EEG. Although apneic seizures originating in the temporal lobe have been observed in term neonates [4], they are not usually associated with changes in heart rate [3]. In fetal lambs, Gunn et al. has shown that during an ischemic event, the EEG becomes isoelectric [8]. Recovery of EEG activity depended on the duration of the ischemic event, with shorter duration events leading to full recovery of EEG activity. If ischemia lasted 30 minutes or longer, a stereotypic sequence of depressed EEG activity followed by low frequency epileptiform activity was always seen. In the newborn piglet model, hypoxic-ischemia induced by reducing fractional inspired oxygen to around 6%, led to rapid suppression of EEG activity. Brain damage was only seen when the EEG amplitude remained suppressed for 23 minutes or more [9]. In another study which exposed one-week-old piglets to graded hypoxia, the EEG amplitude did not decline until oxygen saturation fell below 25%, a similar level at which EEG suppression developed in our neonate [10].

In both episodes in our neonate, bradycardia preceded complete EEG suppression and EEG amplitude did not become profoundly suppressed until oxygen saturation fell below 20%. This is similar to the effects described in animal studies when hypoxia has been used to induce severe EEG suppression [11]. In piglets, EEG amplitude has been shown to decrease markedly after approximately 30 seconds of apnea induced by stimulation of the superior laryngeal nerves [11]. Piglets that were preoxygenated preserved their EEG amplitude during stimulation until the oxygen saturation levels fell below 50%. We believe that hypoxia in conjunction with bradycardia was responsible for the severe EEG suppression in our reported case.

Gavilanes et al. have shown that cerebral neuronal oxygenation is maintained during hypoxia-induced EEG suppression when blood pressure is maintained constantly above 40 mmHg [10]. This suggests that periods of complete EEG suppression during hypoxia may be a neuroprotective mechanism. Animal studies have shown that as soon as cerebral oxygen supply is depleted to a certain critical level, postsynaptic potentials are inhibited by an increase in adenosine (often measured as the breakdown product of hypoxanthine) in the interstitial space via the A1 receptor subtype, resulting in suppression of electrocortical activity [12]. In addition, adenosine may further depress calcium conductance. The actions of adenosine on potassium and calcium metabolism may render the cell less electrically excitable and spare cell energy, avoiding metabolic failure and irreversible cell damage [13]. In rats, immature neurons have been found to be more resistant than adult neurons exposed to hypoxic events. The mechanism for this may be mediated by activation of the N-methyl-D-aspartate receptors or intracellular calcium in the immature brain [13, 14].

Short periods of fetal electrocortical suppression have been reported during labor in humans without any consequences [15]. An adaptive mechanism has been implicated in such short suppression of synaptic transmission activity, where a state of decrease energy requirement is developed to withstand longer hypoxic insults induced by episodes of complete cord occlusion (to mimic uterine contraction in labour) in animal models [16]. In an ovine fetal brain, this adaptive metabolic shutdown appears to be mediated also by endogenous activation of adenosine A1 receptors during critical decreases in oxygenation [17, 18]. The onset of this response has been shown to occur within 30 to 60 seconds after complete cord occlusion in animal models, as measured by a decreased in EEG amplitude or cerebral metabolic rate [17, 18]. 

Using near-infrared spectrometry, a combination of bradycardia and hypoxia has been shown to impair cerebral oxygenation in the human neonate [19], and this may have a role in the pathogenesis of neonatal cerebral injury. Postnatally, it is not known how long apnea or hypoxia can continue before irreparable brain damage occurs. However, it is known that prolonged suppression of electrocortical activity in the neonate is an ominous sign such as that seen following a severe hypoxic-ischemic brain injury. EEG activity may recover but a long recovery period following hypoxic-ischemic injury is associated with an unfavourable long-term neurological outcome [6]. In animal models, EEG suppression following a severe hypoxic-ischemic insult can occur very rapidly and the time required for recovery will depend on the duration and severity of the primary insult [20].

## 4. Conclusion 

Our case report has shown that prolonged apneic episodes accompanied by hypoxia and bradycardia can be associated with altered cerebral function in the neonate. From a clinical perspective, we feel that clinicians would be keen to know the lowest limit of oxygen saturation required to suppress EEG activity. We have shown that not all apneic events are associated with complete EEG suppression, but apneic events with oxygen desaturations below 20% always were. Although complete EEG suppression can be reversible, clinicians should be aware that the recovery from complete EEG suppression depends on the speed of intervention. We have shown that EEG amplitude is exquisitely sensitive to hypoxia and bradycardia in the human neonate.

## Figures and Tables

**Table 1:** Physiological characteristics of apneic events not associated with complete EEG suppressions and those with complete EEG suppressions recorded in the neonate.

	Mean (range)	
	Apnea without complete EEG suppression	Apnea with complete EEG suppression
In relation to apneic episodes		
Number of apneic episodes (*n*)	6	2
Duration of apneic episodes (seconds)	79 (23–119)	First episode = 213 Second episode = 376
In relation to oxygenation desaturation		
Lowest oxygen desaturation (%)	45	First and second episodes <20 (down to unrecordable levels)
Duration of oxygen desaturation (seconds)	137 (72–335)	First episode = 285 Second episode = 361
Lowest oxygen desaturation before complete EEG suppression (%)	—	First episode = 19 Second episode = 4
Duration of oxygen desaturation before complete EEG suppression (seconds)	—	First episode = 55 Second episode = 56
In relation to bradycardia		
Lowest bradycardia (beats per minute)	99	First episode = 66 Second episode = 48
Duration of bradycardia (seconds)	96 (53–224)	First episode = 297 Second episode = 305
Lowest bradycardia before complete EEG suppression (beats per minute)	—	First episode = 66 Second episode = 54
Duration of bradycardia before complete EEG suppression (seconds)	—	First episode = 94 Second episode = 68
In relation to complete EEG suppression		
Number of complete EEG suppression (*n*)	—	2
Duration of complete EEG suppression (seconds)	—	First episode = 68 Second episode = 179
Recovery time from oxygen desaturation after complete EEG suppression ended (seconds)	—	First episode = 51 Second episode = 129
